# Honey from Afyonkarahisar (Türkiye) exhibits low toxic-element burdens and distinct spatial elemental patterns

**DOI:** 10.1038/s41598-026-55009-7

**Published:** 2026-06-03

**Authors:** Ömer Gümüştaş, Ulas Acaroz

**Affiliations:** 1 Institute of Health Sciences, Department of Food Hygiene and Technology, Afyon Kocatepe University, Afyonkarahisar, 03200 Türkiye; 2 Faculty of Veterinary Medicine, Department of Food Hygiene and Technology, Afyon Kocatepe University, Afyonkarahisar, Türkiye; 3 Faculty of Veterinary Medicine, Department of Food Hygiene and Technology, Kyrgyz-Turkish Manas University, Bishkek, Kyrgyzstan

**Keywords:** Honey, Elemental fingerprinting, Toxic elements, Spatial variability, Dietary risk, Afyonkarahisar (Türkiye), Biomarkers, Cancer, Environmental sciences, Risk factors

## Abstract

Honey integrates environmental inputs over the foraging range of honeybees, making it both a food and a natural sampler of local elemental exposure. Across 40 honey samples from 19 apiaries, Afyonkarahisar honeys displayed distinct multi-element compositions shaped by both regional background and localized enrichment. The mineral fraction was dominated by potassium, while iron and zinc were the most abundant trace elements. Toxic elements were consistently low: lead averaged 0.0051 mg kg⁻¹ (maximum 0.0346 mg kg⁻¹; below the commonly cited 0.10 mg kg⁻¹ level for honey), with similarly low concentrations of cadmium, mercury and arsenic. Multivariate analyses resolved two main compositional groups and four sub-groups with distinct elemental signatures, reflecting a gradient from low overall mineralization to localized multi-element enrichment. At typical honey consumption levels (30 g day⁻¹ for adults; 20 g day⁻¹ for children), all target hazard quotients were < 1 and the combined hazard index remained low (HI 0.0366 for adults; 0.0853 for children). Lifetime cancer risk estimates for Cr, Ni, As and Cd were 7.15 × 10⁻⁵ (adults) and 1.67 × 10⁻⁴ (children), within or close to commonly used tolerability ranges. Overall, Afyonkarahisar honeys appear safe for consumers and provide element fingerprints capable of resolving fine‑scale spatial variability in environmental elemental signatures.

**Supplementary Information:**

The online version contains supplementary material available at 10.1038/s41598-026-55009-7.

## Introduction

What insights do the elemental fingerprints in honey provide about local environmental conditions and human exposure to elements through a widely consumed natural product? Honey is produced by bees that forage across a limited landscape around the hive, integrating signals from soils, plants, dust and water over the flowering season. This makes honey more than a nutritious food: it can also function as a practical bioindicator matrix for assessing environmental contamination patterns and their potential implications for consumers^1,13^.

Honey’s value as a food and traditional remedy is linked to its complex composition, including sugars, phenolic compounds, organic acids, enzymes and small peptides, which recent reviews associate with antioxidant, antimicrobial and anti-inflammatory activity^2,3^. From an environmental and geochemical viewpoint, however, the key feature is its small but informative mineral fraction. The mineral fraction of honey is generally dominated by macrominerals—particularly potassium, sodium, calcium and magnesium—accompanied by trace elements such as iron, manganese, zinc, copper and selenium^4,5^. While these elements contribute to nutritional value and quality characteristics, the same environmental pathways can also introduce non-essential and toxic elements (e.g., lead, cadmium, arsenic and mercury), which have no physiological function and are linked to adverse outcomes under chronic exposure^6–8^.

Element levels in honey reflect the combined influence of natural background and anthropogenic inputs. Background patterns are shaped by geogenic and atmospheric processes, whereas traffic emissions, industrial activities and intensive agricultural practices can increase element loading in soils, dust and surface waters^6^. Elements can then be transferred through soil–plant interactions into nectar and honeydew and, ultimately, into honey. Studies support this environmental linkage, reporting higher levels of certain elements in honeys sampled from more impacted environments compared with less impacted settings, consistent with local contamination pressure and habitat conditions^9–12^.

This integration across the foraging landscape is the basis for using bees and hive products—honey included—as bioindicators in environmental monitoring. A dedicated review has synthesized evidence supporting the use of honeybees and honey as bioindicators of element contamination^13^. More recent targeted studies have further demonstrated how hive products can be applied to assess environmental contamination in specific regions, including southern Italy^1^. Large-scale and regional datasets also suggest that, even when overall concentrations are low, multi-element patterns in honey can still reveal geographically structured signatures consistent with differing environmental and land-use settings^15–17^. In this context, elemental fingerprints are informative not only for food-safety screening, but also for identifying localized enrichment signals that may warrant environmental attention.

Beyond bioindication, multi-element profiles provide a chemical basis for geographical characterisation. ICP-based studies frequently combine macro/trace/toxic element data with chemometric tools (e.g., principal component analysis and cluster analysis) to distinguish honey groups and to highlight how local environmental conditions shape compositional structure^5,9,10,18^. Across regions, potassium is commonly reported as the dominant macromineral and iron, manganese and zinc are often among the most abundant trace elements, while absolute concentrations vary with local settings and pressure gradients^4,5^. Importantly, chemometric grouping can help separate broad background variability from smaller subsets of samples showing enrichment patterns.

Because honey is also directly consumed, contaminant measurement is increasingly paired with health risk assessment to translate concentrations into exposure-relevant metrics. Many studies estimate dietary exposure and non-carcinogenic risk using estimated daily intake and hazard indices, often finding hazard indices below unity for typical intake scenarios while noting the importance of consumption level, particularly for children^7,10,19^. Newer frameworks also propose integrative approaches for contextualising combined element exposure in honey^20^. This risk-based perspective is central to the bioindicator question because it connects environmental signals in honey to potential implications for human exposure.

Türkiye is a major honey-producing country with a strong apiculture tradition^14^. ICP-based surveys of Turkish honeys commonly report potassium as the predominant element and identify iron, zinc, manganese and copper among the main trace elements, with toxic elements generally low but measurable^21,22^. Afyonkarahisar province includes diverse districts and land-use settings that could plausibly generate heterogeneous elemental signatures in honey. However, to the best of our knowledge, Afyonkarahisar honeys have not yet been systematically evaluated as an elemental bioindicator dataset that simultaneously addresses (i) multi-element structure and spatial/chemometric grouping and (ii) consumer exposure and risk.

Accordingly, this study asks: What do the multi-element fingerprints in Afyonkarahisar honeys reveal about local environmental variability and human exposure to elements? To address this question, we aimed to (i) quantify selected macroelements, essential trace elements and toxic elements in honeys from multiple apiaries in Afyonkarahisar using ICP–MS; (ii) examine compositional relationships using correlation analysis and chemometric methods (PCA and hierarchical clustering) to identify dominant patterns and potential localized enrichment; and (iii) estimate dietary exposure and non-carcinogenic/carcinogenic risks for adults and children using widely applied risk-assessment metrics. By aligning bioindicator interpretation with exposure estimation, this work provides a geochemical and toxicological characterisation of Afyonkarahisar honeys and supports their use in regional environmental monitoring.

##  Materials and methods

**Fig. 1:**
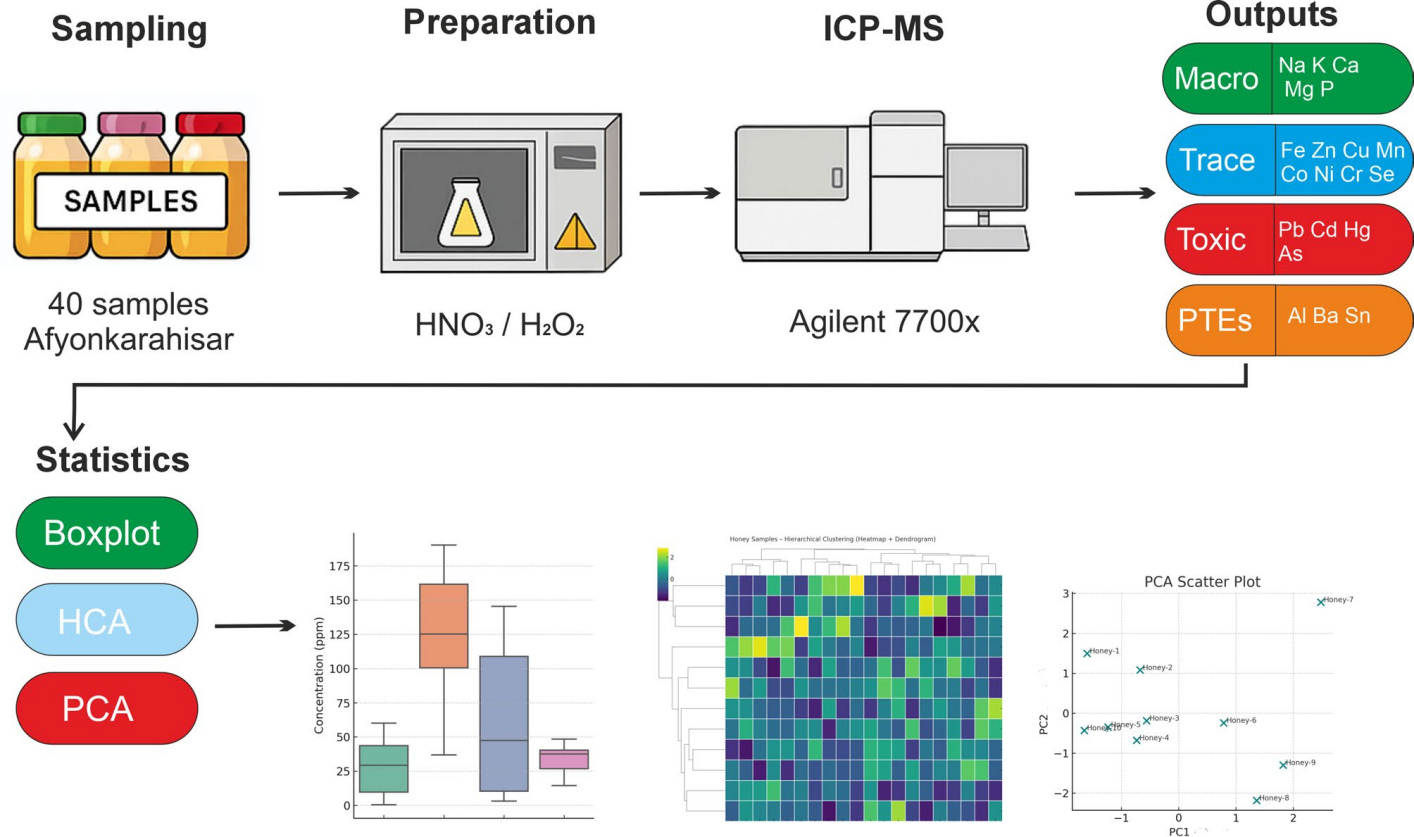
Overall experimental workflow of the study. Forty honey samples collected from Afyonkarahisar were digested using HNO₃/H₂O₂ and analysed by ICP-MS (Agilent 7700x). The obtained elemental data (macro, trace, potentially toxic (PTEs), and toxic elements) were evaluated using boxplot, hierarchical cluster analysis (HCA), and principal component analysis (PCA).

The overall experimental workflow of the study, including sample collection, microwave digestion, ICP–MS analysis, and chemometric evaluation, is shown in Fig. 1.

### Ethical statement

The present study was reviewed by the Local Ethics Committee for Animal Experiments of Afyon Kocatepe University (AKÜHADYEK). According to the official decision (Ref. No: AKÜHADYEK-71-18), this research does not require ethical approval. Because it involves only honey sample collection and no animal experimentation. All samples were collected from local beekeepers with their consent and handled in accordance with national and institutional guidelines for food and environmental research.

### Study area and sample collection

**Fig. 2:**
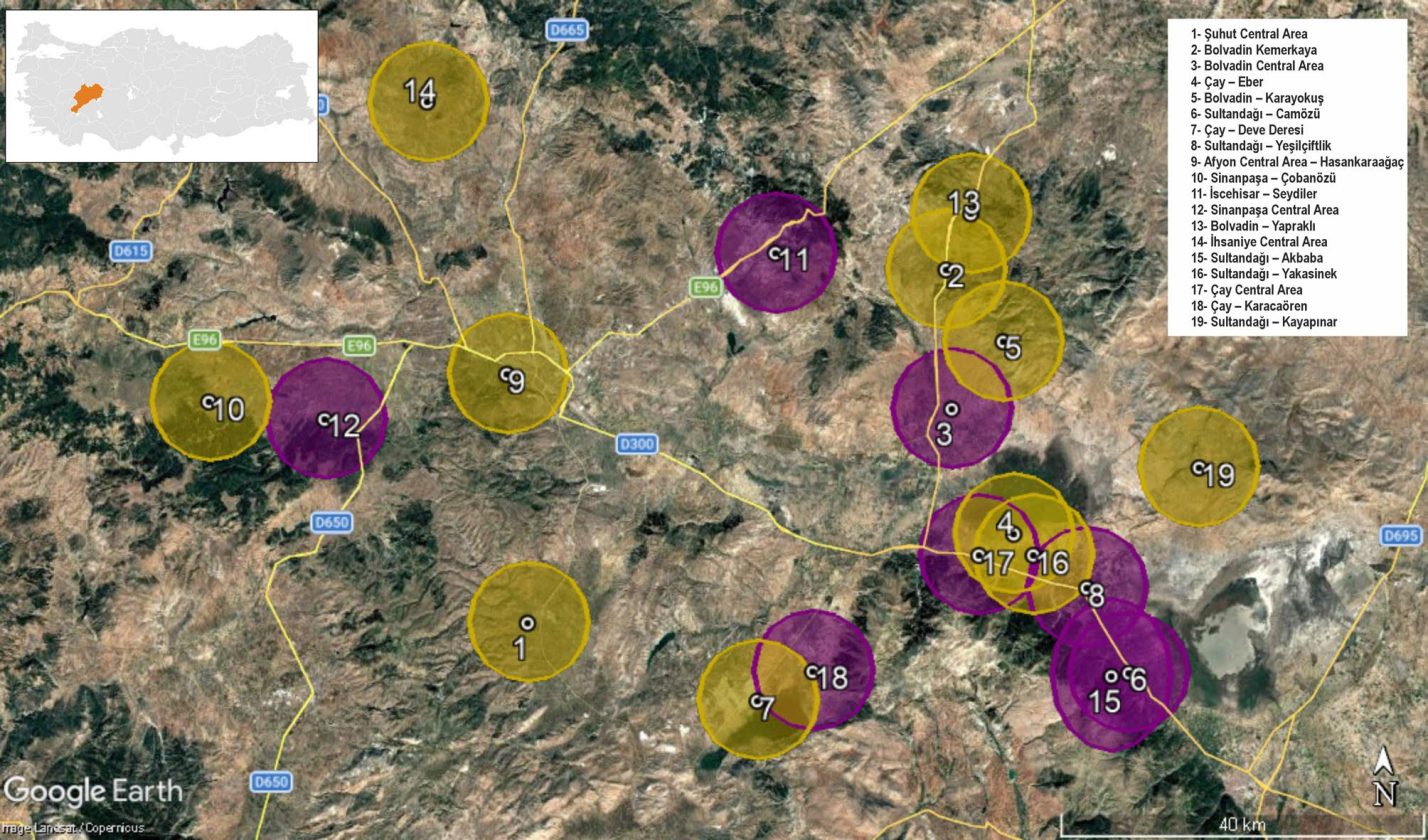
Distribution map of the sampling sites in Afyonkarahisar Province, Türkiye. Each numbered circle represents one sampling location and the 13 km radius around the central point indicates the approximate honeybee foraging buffer (apiary zone). Sampling locations are color-coded according to the two main groups identified by hierarchical cluster analysis (Ward.D2, k = 2): Main Group A (purple; lower overall mineralization) and Main Group B (gold; higher overall mineralization). The internal sub-structure of these groups is detailed in Fig. 8. Numbers correspond to the sampling-location list shown on the right. Map source: Google Earth Pro (2020; imagery © Google, Landsat/Copernicus). All circles, boundaries, and annotations were created by the authors using Google Earth Pro.

A total of 40 honey samples were collected from 19 apiary locations within the central and district boundaries of Afyonkarahisar province (Western Türkiye) during the 2019–2020 honey season (Fig. 2). The sampling area represents a region characterized by a mixed floral composition (including both wild and cultivated plant sources) and a moderate continental climate.

Detailed information on the sampling locations, including location codes, corresponding sample numbers, geographic coordinates, and site names, is provided in Table S1. The spatial distribution of the apiaries covered both central urban zones and rural districts, allowing for representative sampling across the study area.

Each sample (approximately 250 mL) was obtained directly from local beekeepers, placed into pre-cleaned polyethylene containers, properly labeled, and stored at room temperature until analysis.

### Sample preparation

Honey samples were digested using an Ethos Easy microwave digestion system (Milestone, Italy). The digestion procedure was based on the standard method for honey mineral analysis. Approximately 0.500 g of each sample was accurately weighed into 100 mL Teflon (TFM) microwave digestion vessels. Then, 9 mL of suprapure nitric acid (HNO₃ 65 w w⁻¹) and 1 mL of hydrogen peroxide (H₂O₂ 30 w w⁻¹) were added to each vessel, which was subsequently sealed. The microwave program was set to reach 180 °C within 10 min, maintained at this temperature for an additional 10 min, and then allowed to cool down to room temperature. After digestion, the solutions were filtered through 0.45 μm PTFE filters and diluted to the appropriate volume with ultrapure water prior to analysis.

### Elemental analysis by ICP–MS

Elemental quantification was carried out using an Agilent Technologies 7700 ICP–MS system (Agilent Technologies, Tokyo, Japan). The instrument was equipped with a collision/reaction cell and operated in helium (He) mode to minimize polyatomic interferences. The following isotopes were monitored for quantification: ²⁷Al, ⁷⁵As, ¹³⁷Ba, ⁴⁴Ca, ¹¹¹Cd, ⁵⁹Co, ⁵²Cr, ⁶³Cu, ⁵⁶Fe, ²⁰¹Hg, ³⁹K, ²⁴Mg, ⁵⁵Mn, ²³Na, ⁶⁰Ni, ³¹P, ²⁰⁸Pb, ⁷⁸Se, ¹¹⁸Sn, ⁶⁶Zn. Operating conditions included a nebulizer gas flow of 0.9 L min⁻¹, auxiliary gas flow of 0.9 L min⁻¹, plasma gas flow of 15.0 L min⁻¹, RF power of 1500 W, sampling depth 8.0 mm, Kinetic energy discrimination 3 V, spray chamber temperature 2 °C. ^45^Sc, ^103^Rh, ^115^In were used as internal standards. Quantification was performed based on calibration curves which showed excellent linearity (R² > 0.995). Results were expressed as mg kg⁻¹ on a fresh weight basis. Limits of detection (LOD) and quantification (LOQ) were established using signal-to-noise criteria (LOD = S/N 3; LOQ = S/N 10) and varied among elements (Table S2). LOD values ranged from 0.00002 to 0.01917 mg kg⁻¹, while LOQ values ranged from 0.00006 to 0.06390 mg kg⁻¹. Method accuracy was evaluated using the certified reference material NIES No.7 (Tea Leaves, NIES, Japan) and matrix spike recovery tests; analytical precision is presented in Table S2.

### Element classification

According to their biological roles and toxicity, the analysed elements were categorized into four groups:

- Macro elements: Na, K, Ca, Mg, P (Nutritional and botanical origin).
- Trace elements: Fe, Zn, Cu, Mn, Co, Ni, Cr, Se (Biologically relevant trace minerals).
- Potentially toxic elements (PTEs): Al, Ba, Sn (Non-essential elements with possible toxic effects at high levels).
- Toxic elements: Pb, Cd, Hg, As (Elements with no known biological function and high toxicity).

### Health risk assessment

Human exposure to elements through honey consumption was evaluated using standard US EPA approaches for non‑carcinogenic and carcinogenic risk. For each element, the estimated daily intake (EDI, mg kg⁻¹ bw day⁻¹) was calculated as: EDI = (C × IR)/BW, where C is the concentration in honey (mg kg⁻¹), IR is the ingestion rate (0.03 kg day⁻¹ for adults and 0.02 kg day⁻¹ for children), and BW is body weight (70 kg for adults and 20 kg for children). Non‑carcinogenic risk was expressed as the target hazard quotient (THQ) and hazard index (HI): THQ = EDI/RfD, where RfD is the oral reference dose (mg kg⁻¹ day⁻¹), and HI = ΣTHQ across elements. RfD values were selected based on previous literature for Al, As, Co, Cu, Fe, Hg, Mn, Ni, Pb, Zn^23^, Cd, Cr, Ba, Se^24^. THQ or HI values < 1 indicate that adverse non‑carcinogenic effects are unlikely. The RfD values and oral slope factors used in these calculations are summarized in Table S3. Carcinogenic risk (CR) was estimated for elements based on the formula: CR = EDI × SF. Slope factors were selected based on previous studies for Cr, Ni, As^25^ and Cd^26^. Following common practice, total lifetime cancer risk (CR_total) in the range of 10⁻⁶–10⁻⁴ was considered acceptable/tolerable.

### Statistical analysis

All statistical analyses and figures were produced in R (v4.5.1). Elemental concentrations (mg kg⁻¹) were summarized using descriptive statistics (min–max, mean, SD, median, IQR and coefficient of variation) and visualized with boxplots. Prior to multivariate analyses, concentration data were log1p‑transformed and z‑score standardized.

Relationships among elements were assessed using Spearman’s rank correlation (ρ) with two‑sided p‑values. Principal component analysis (PCA) was performed to explore multivariate structure, and results were visualized as a biplot (PC1–PC2). Hierarchical cluster analysis (HCA) was conducted using Euclidean distance and Ward’s D2 linkage and displayed as a dendrogram and clustered heatmap; the number of descriptive sub-groups was guided by silhouette analysis.

## Results

**Fig. 3:**
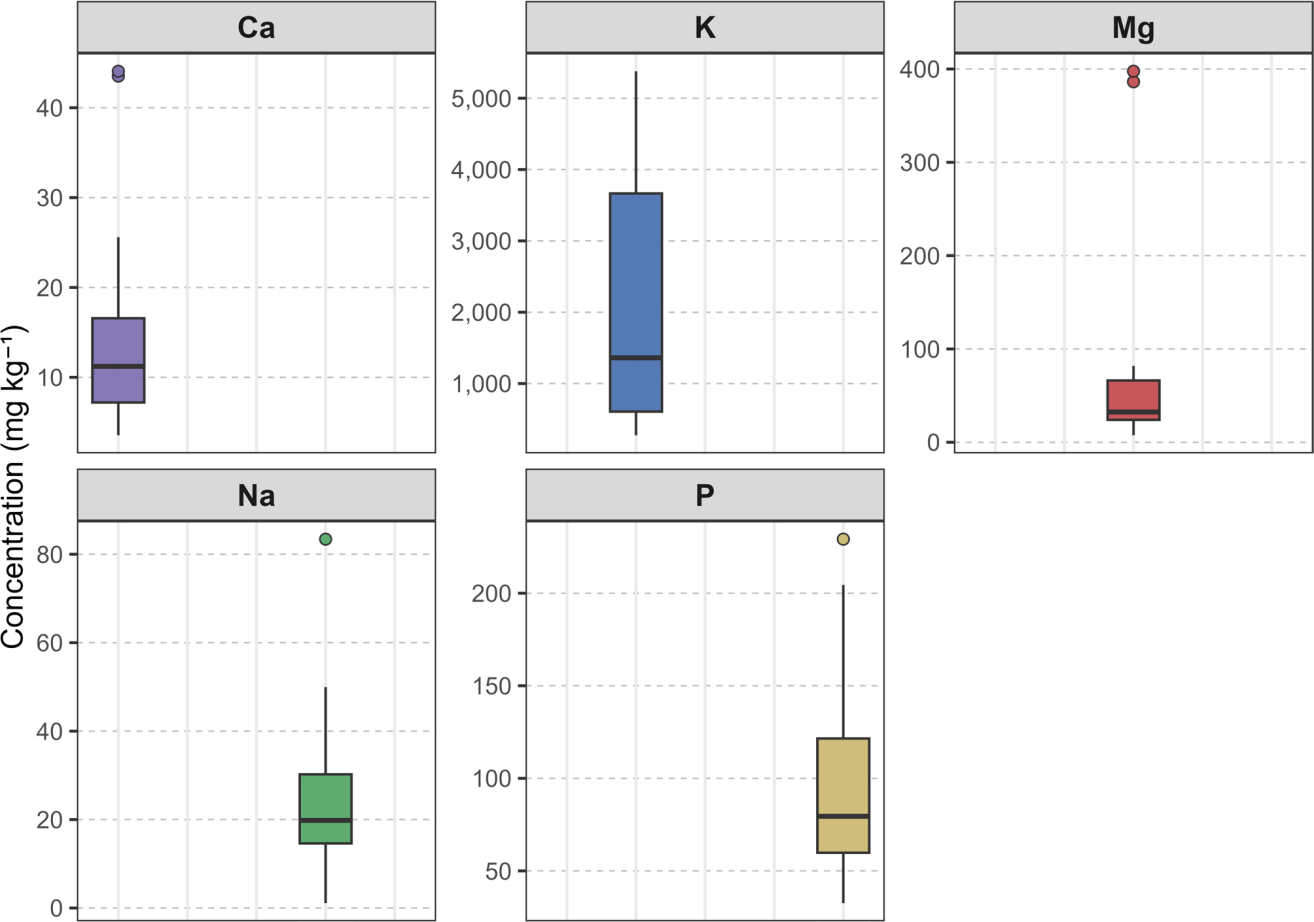
Macro elements in honey samples. Boxplots of K, Ca, Mg, Na, and P concentrations in honey samples (*n* = 40), reported as mg kg⁻¹.

**Fig. 4:**
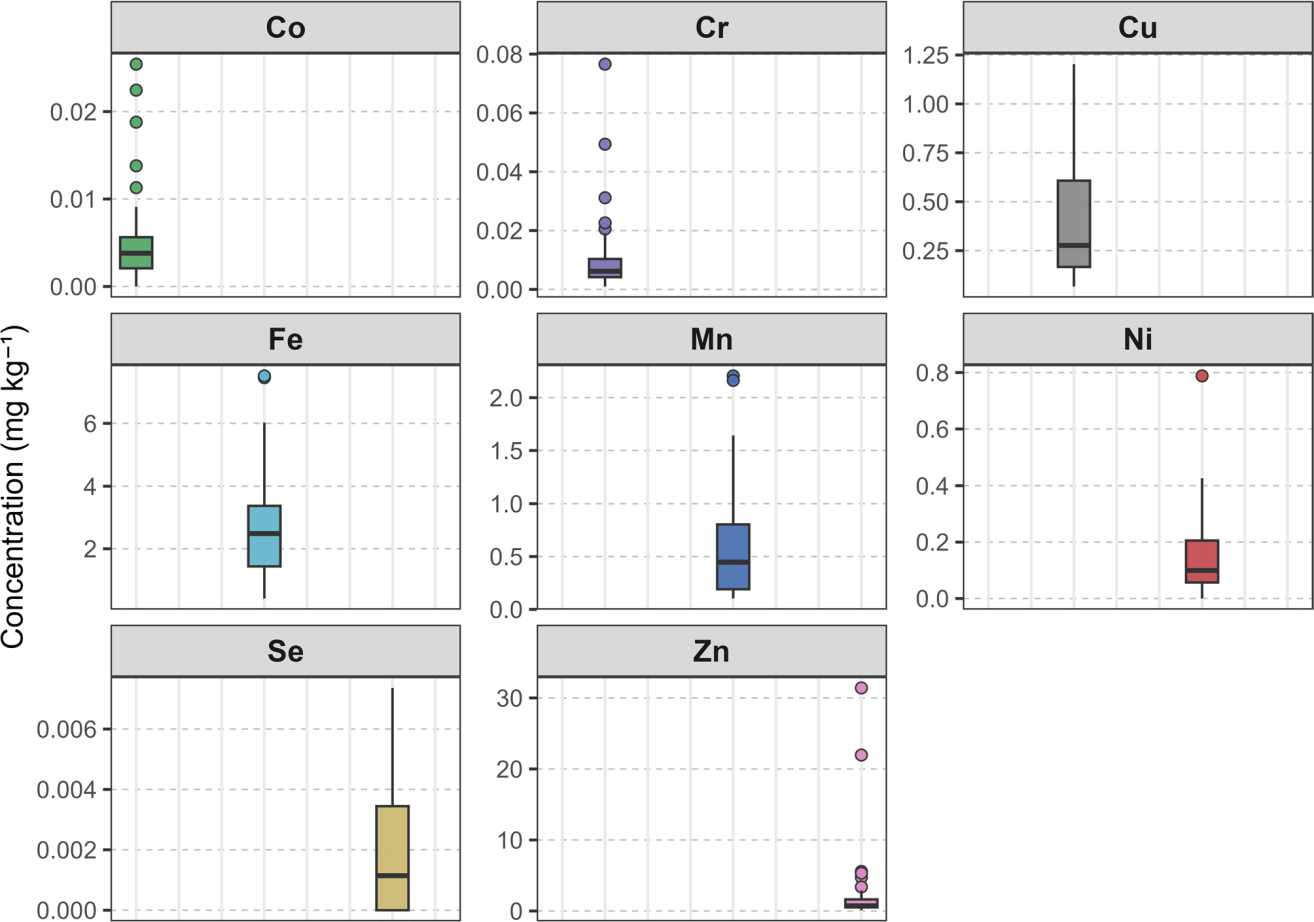
Trace elements in honey samples. Boxplots of Co, Cr, Cu, Fe, Mn, Ni, Se, and Zn concentrations in honey samples (*n* = 40), reported as mg kg⁻¹.

**Fig. 5:**
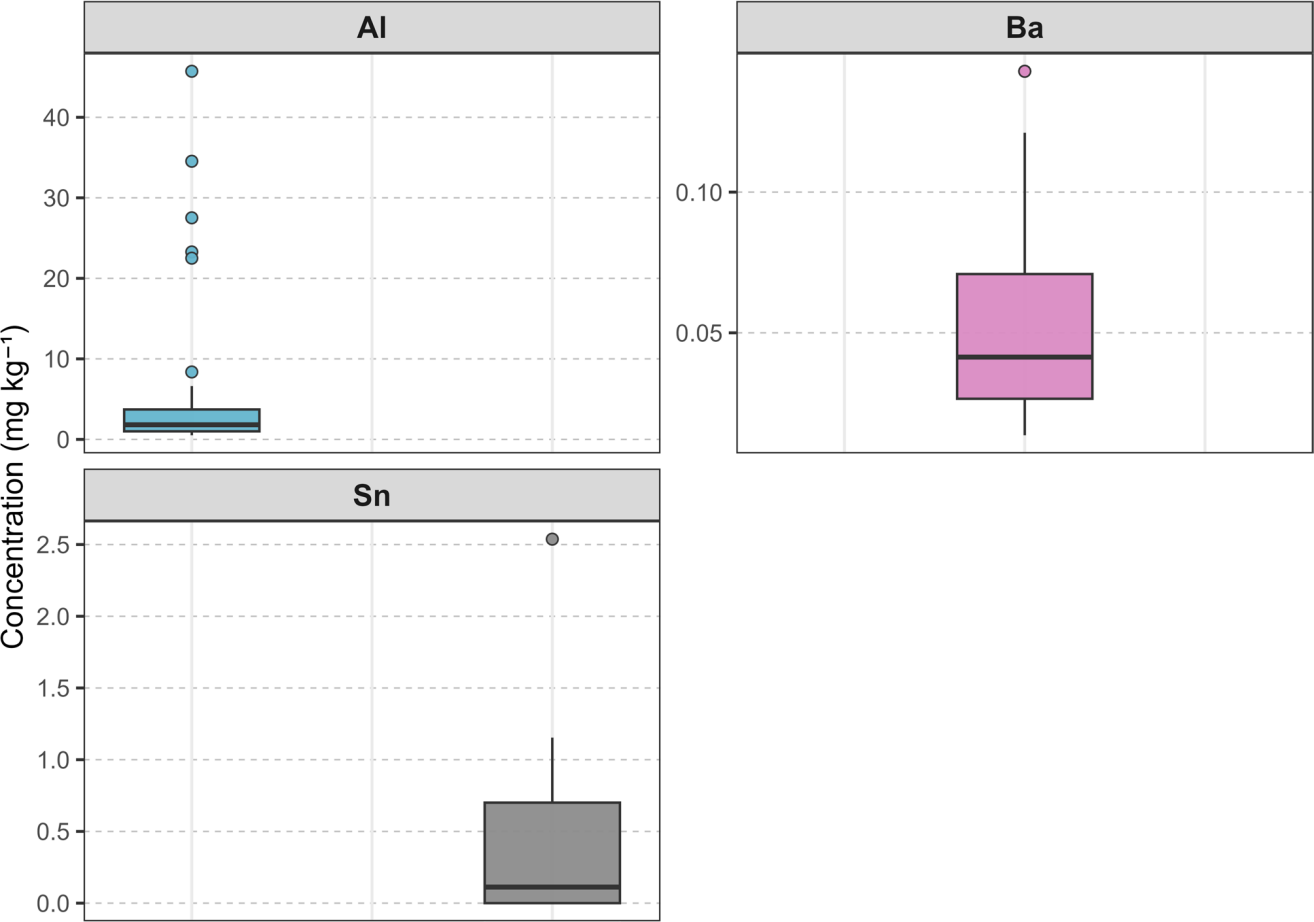
Potentially toxic elements in (PTEs) honey samples. Boxplots of Al, Ba, and Sn concentrations in honey samples (*n* = 40), reported as mg kg⁻¹.

**Fig. 6:**
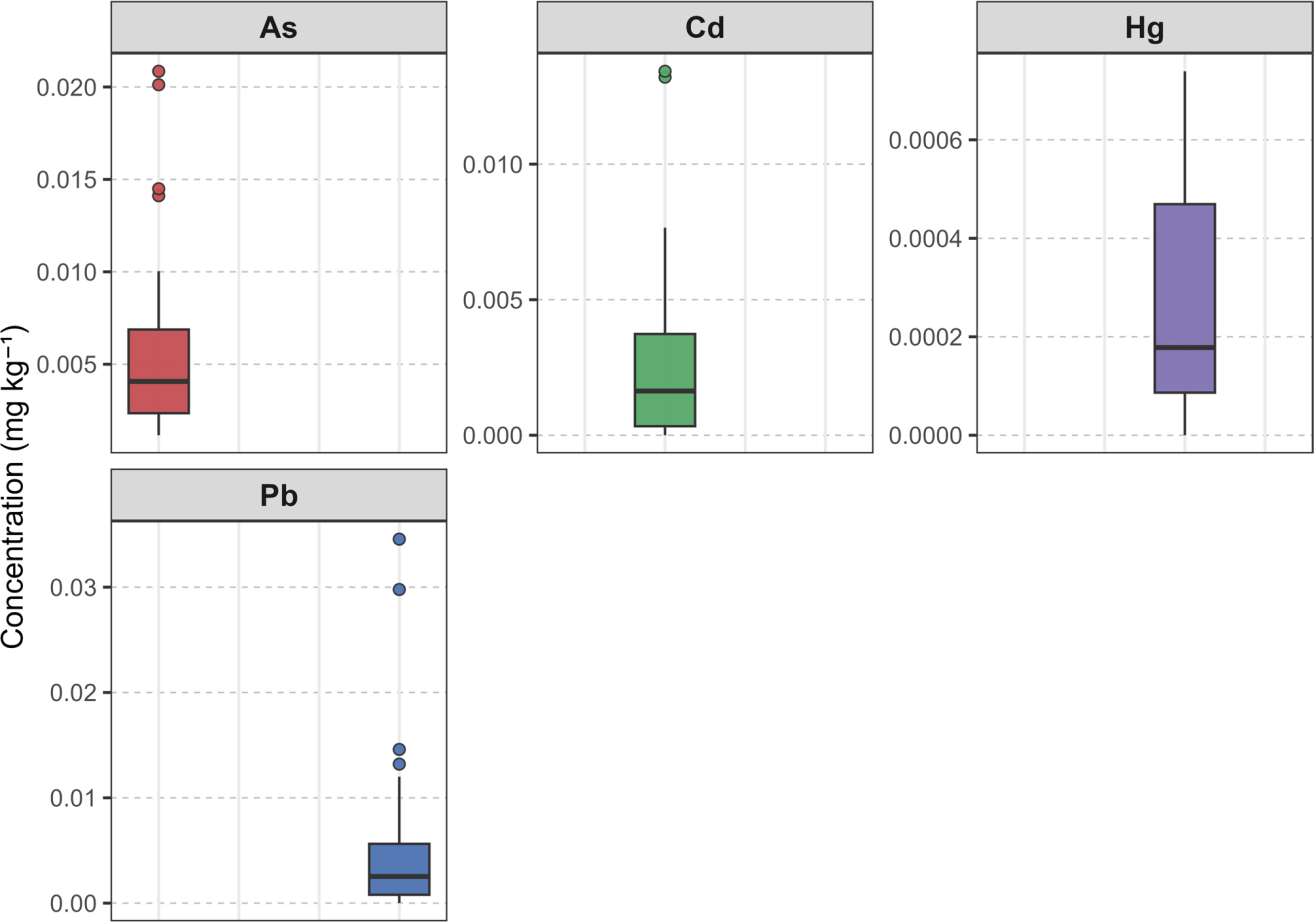
Toxic elements in honey samples. Boxplots of As, Cd, Hg, and Pb concentrations in honey samples (*n* = 40), reported as mg kg⁻¹.

Descriptive statistics for all elements are provided in Table S4, and the full Spearman correlation matrix is shown in Fig. S1 Boxplots summarizing element distributions are presented in Figs. 3, 4, 5 and 6, while sample‑wise stacked bar charts are provided as Fig. S2–S5.

### Macro elements (Na, K, Ca, Mg, and P)

Macroelement concentrations were dominated by K (mean 2195.59 mg kg⁻¹; range 275.22–5377.37 mg kg⁻¹), which also showed substantial between-sample variability (CV 80.06%). P was the next most abundant macroelement (mean 92.81 mg kg⁻¹; 32.58–229.20 mg kg⁻¹), followed by Mg (mean 57.31 mg kg⁻¹; 7.51–397.57 mg kg⁻¹). In contrast, Na (mean 23.19 mg kg⁻¹; 1.11–83.41 mg kg⁻¹) and Ca (mean 13.60 mg kg⁻¹; 3.55–44.06 mg kg⁻¹) occurred at lower levels. Distributions (Fig. 3) indicate marked right-skewness for K and Mg, consistent with a limited number of high-value samples, whereas Na and Ca were comparatively more compact; sample-wise patterns are shown in Supplementary Fig. S2.

Correlation analysis supported this pattern: K, Mg and P co-varied strongly (ρ = 0.91–0.93), while Ca showed only weak associations with the remaining macroelements (Fig. S1).

### Trace elements (Fe, Zn, Cu, Mn, Co, Ni, Cr, and Se)

Among trace elements, Fe and Zn had the highest mean concentrations (Fe: mean 2.71 mg kg⁻¹; 0.41–7.52 mg kg⁻¹; Zn: mean 2.55 mg kg⁻¹; 0.11–31.41 mg kg⁻¹). Zinc displayed the greatest dispersion (CV 230.55%), reflecting a small number of enriched samples (Fig. 4). Cu (mean 0.40 mg kg⁻¹; 0.07–1.20 mg kg⁻¹) and Mn (mean 0.61 mg kg⁻¹; 0.10–2.21 mg kg⁻¹) were present at intermediate levels, whereas Co, Ni, Cr and Se occurred at generally low concentrations with high relative variability across samples. Sample-wise distributions are provided in Fig. S3.

Spearman correlations highlighted a coherent Cu–Mn–Ni association (Cu–Mn ρ = 0.89; Cu–Ni ρ = 0.77; Mn–Ni ρ = 0.73). In addition, Fe was positively related to Mn, Ni and Cr (ρ ≈ 0.65–0.72), and Cr showed a positive association with Zn (ρ = 0.61), while Se exhibited weak relationships with most elements (Fig. S1).

### Potentially toxic elements (Al, Ba, and Sn)

Among potentially toxic elements, Al showed the highest concentrations and the strongest variability (mean 5.83 mg kg⁻¹; 0.52–45.71 mg kg⁻¹; CV 174.42%) (Fig. 5). Sn was lower on average (mean 0.39 mg kg⁻¹) but ranged up to 2.54 mg kg⁻¹, indicating sporadic enrichment in some samples. Ba remained consistently low (mean 0.0546 mg kg⁻¹; 0.0136–0.1429 mg kg⁻¹). Sample-wise patterns are shown in Fig. S4.

Al tended to increase together with several macro/trace elements (e.g., K, Mg, P, Mn, Cu and Ni; ρ ≈ 0.71–0.88), whereas Ba showed its clearest positive association with Ca (ρ = 0.64). Sn generally displayed negative relationships with several transition elements (e.g., Co and Ni) (Fig. S1).

### Toxic elements (Pb, Cd, Hg, and As)

Toxic elements were detected at low levels overall (Fig. 6). Concentrations ranged from 0.0012 to 0.0209 mg kg⁻¹ for As (mean 0.0056 mg kg⁻¹), 0–0.0134 mg kg⁻¹ for Cd (mean 0.0029 mg kg⁻¹), 0–0.0007 mg kg⁻¹ for Hg (mean 0.0003 mg kg⁻¹), and 0–0.0346 mg kg⁻¹ for Pb (mean 0.0051 mg kg⁻¹). Distributions were typically right-skewed, indicating that most samples contained very low levels with a limited number of higher observations; sample-wise patterns are provided in Fig. S5.

Within this group, the clearest association was observed between Cd and Hg (ρ = 0.57) and, to a lesser extent, Cd and Pb (ρ = 0.37), while As showed weak correlations with the other toxic elements (Fig. S1).

### Principal component analysis (PCA)

**Fig. 7:**
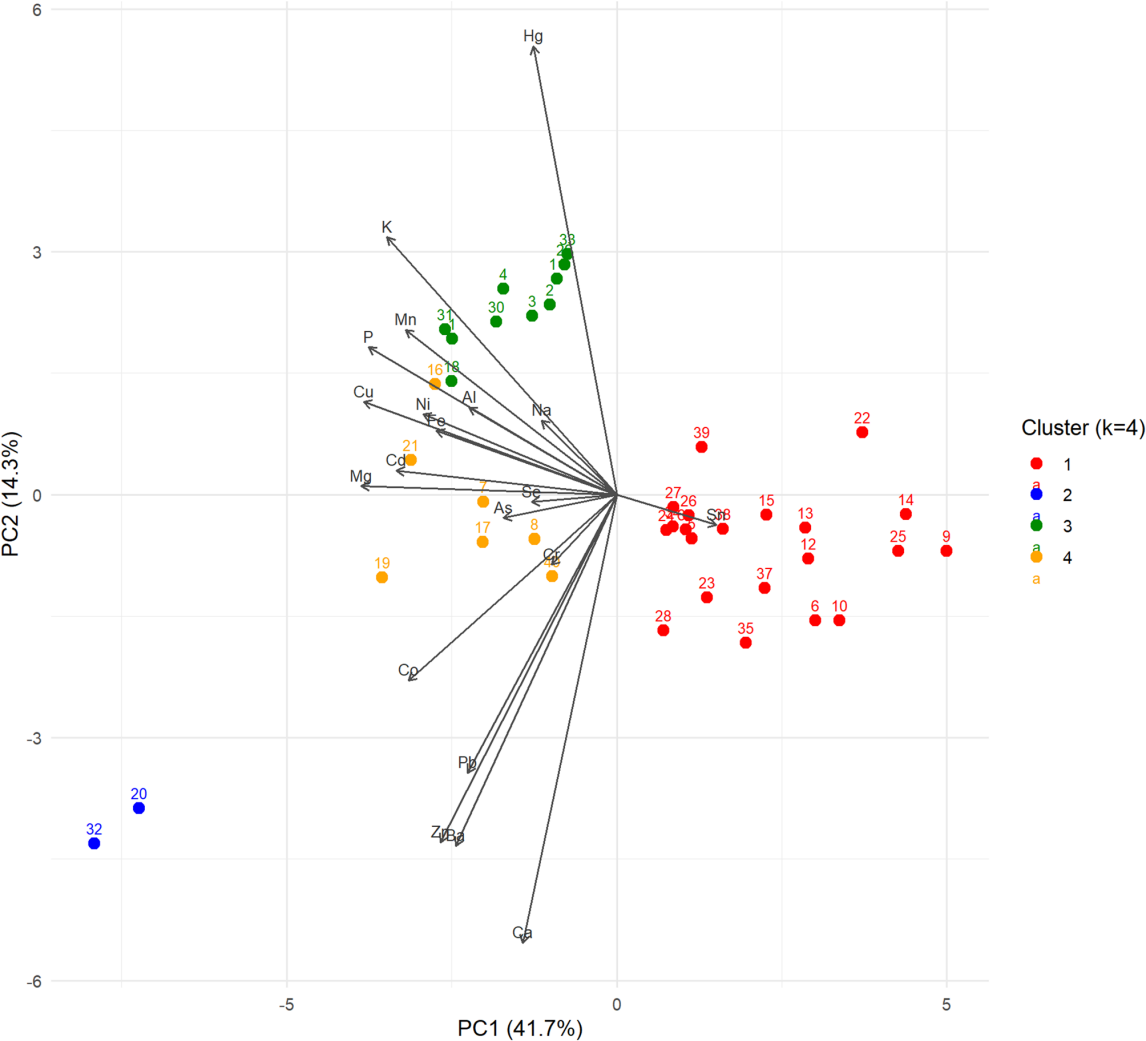
Principal Component Analysis (PCA) biplot of elemental composition in honey samples. Samples are coloured by Ward.D2 sub-group membership (k = 4): Sub-group 1 (red; *n* = 21, low overall mineralization), Sub-group 2 (blue; *n* = 2, samples 20 and 32), Sub-group 3 (green; *n* = 10, moderate mineralization with elevated Hg), and Sub-group 4 (orange; *n* = 7, elevated Al, Mn and Fe). Element loading arrows indicate the direction and strength of each variable’s contribution to PC1 (41.7%) and PC2 (14.3%).

Principal component analysis (PCA) was performed on the log1p‑transformed and z‑score standardized elemental dataset to examine multivariate compositional patterns among honey samples (Fig. 7). The first two principal components explained 56.9% of the total variance (PC1: 41.7%, PC2: 14.3%), while the first three components accounted for 66.1%. Most samples clustered near the center of the score space, indicating broadly comparable multi‑element profiles. Samples 20 and 32 were clearly separated along both PC1 and PC2, while samples 9, 14, 22 and 25 occupied the opposite end of PC1, reflecting comparatively low overall elemental concentrations. These peripheral positions are consistent with the four sub-groups identified by HCA (Fig. 7, colour-coded by k = 4 membership). Based on loading magnitudes, variability along PC1 was mainly associated with Mg, Cu, P, K, Cd and Mn, whereas PC2 captured a contrast driven primarily by Hg and Ca, with additional contributions from Ba, Zn and Pb (Table S5).

### Hierarchical cluster analysis (HCA)

**Fig. 8:**
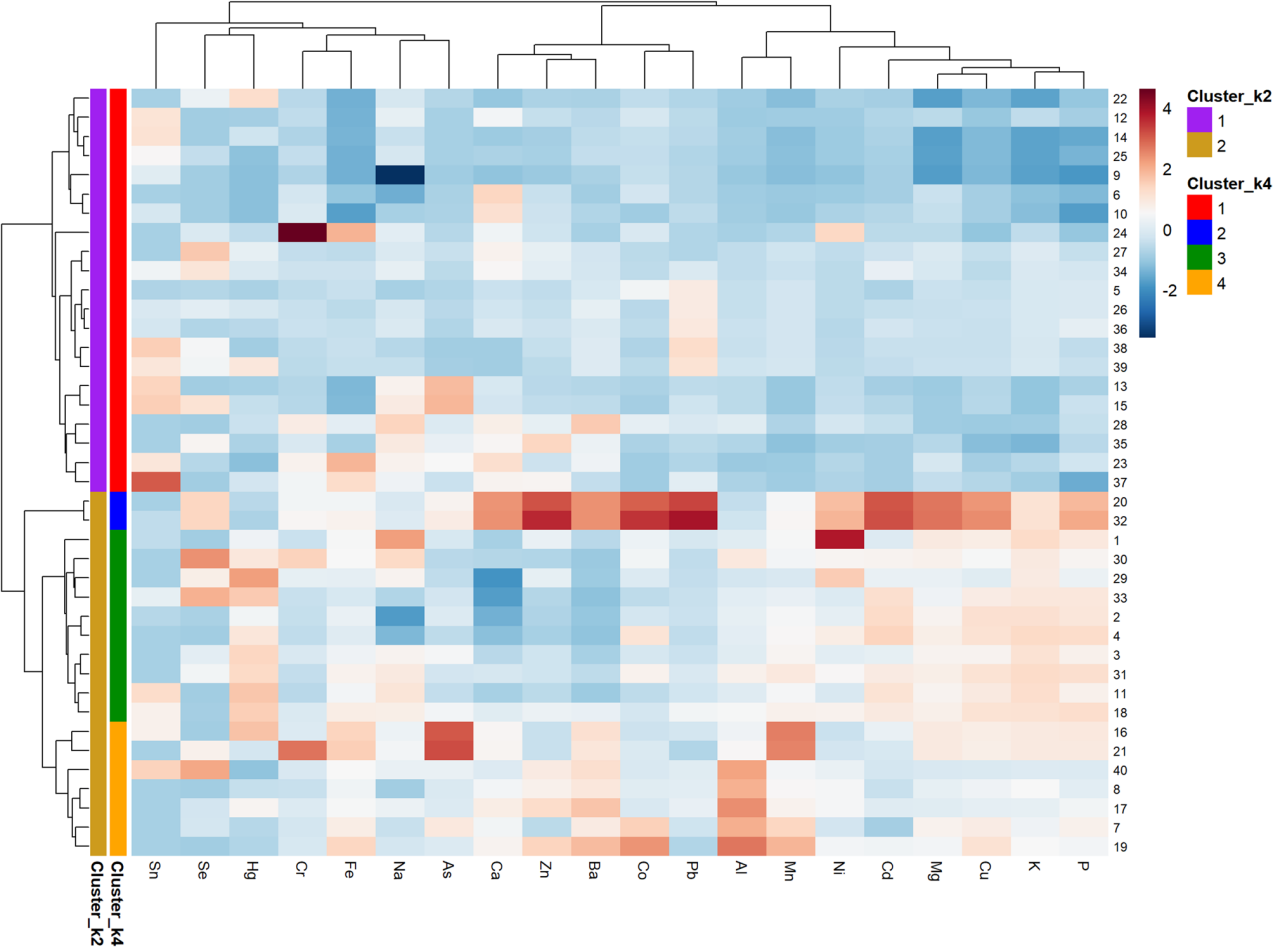
Hierarchical cluster analysis (HCA) heatmap of log1p-transformed and z-score standardized elemental concentrations in honey samples (Ward.D2, Euclidean distance). Row annotation bars indicate the two main groups (k = 2; purple and gold, matching Fig. 2) and the four sub-groups (k = 4; red, blue, green, orange, matching Fig. 7). Colour scale represents standardized element concentrations (blue = below average, red = above average).

Hierarchical cluster analysis (HCA) using Euclidean distance and Ward’s D2 linkage was applied to the same pre‑processed dataset (Fig. 8). Silhouette analysis yielded comparable widths for k = 3 and k = 4 (0.311 and 0.310, respectively; Fig. S6a); the dendrogram was therefore cut at k = 4 to provide a more detailed sub-group characterization (Fig. S6b), yielding four sub-groups within two main hierarchical branches (Fig. 8, Table S6).

Main Group A (Sub-group 1; *n* = 21) displayed consistently negative mean z-scores across nearly all elements, representing the low-mineralization baseline of the dataset. Within this group, samples 9, 12, 14, 22 and 25 formed a distinct sub-branch with particularly low elemental levels, consistent with their peripheral position on the positive end of PC1 (Fig. 7). Main Group B comprised three sub-groups with progressively higher overall elemental levels: Sub-group 3 (*n* = 10) showed moderate enrichment led by Hg, K and P; Sub-group 4 (*n* = 7) was distinguished by elevated Al, Mn and Ba, suggesting a soil/dust-related input; and Sub-group 2 (*n* = 2; samples 20 and 32) exhibited the strongest multi-element enrichment, most notably in Pb, Zn, Co and Cd together with elevated Mg, Ba, Cu and Ca (Table S7).

### Estimated daily intake (EDI) and health risk assessment

The EDIs of all evaluated elements, calculated for a daily consumption of 30 g honey for adults and 20 g for children, were substantially lower than the corresponding oral reference doses (Table 1). For adults, individual THQ values ranged from 1.2 × 10⁻⁴ (Ba) to 7.9 × 10⁻³ (As), while for children they ranged from 2.7 × 10⁻⁴ (Ba) to 1.9 × 10⁻² (As). Although Co and As contributed the highest THQs in both age groups, all THQ values remained far below 1. The cumulative hazard index (HI) was 0.0366 for adults and 0.0853 for children, indicating no appreciable non‑carcinogenic risk under the assumed intake scenario.

Carcinogenic risks were calculated for Cr, Ni, As and Cd. For adults, individual CR values ranged from 2.36 × 10⁻⁶ (Cr) to 5.81 × 10⁻⁵ (Ni), yielding CR_total = 7.15 × 10⁻⁵. For children, CR values ranged from 5.50 × 10⁻⁶ (Cr) to 1.36 × 10⁻⁴ (Ni), with CR_total = 1.67 × 10⁻⁴, which is close to the upper bound of the commonly used acceptable range (10⁻⁶–10⁻⁴). These estimates reflect conservative default assumptions (e.g., speciation not considered) and suggest low carcinogenic risk attributable to honey consumption at the evaluated intake levels.

**Table 1:** Non-carcinogenic (THQ, HI) and carcinogenic (CR) risk estimates for adults and children based on elemental concentrations in Afyonkarahisar honeys.

Element	Mean (mg kg⁻¹)	THQ (Adult)	THQ (Child)	CR (Adult)	CR (Child)
Cr	0.010997	0.001571	0.003666	2.36 × 10⁻⁶	5.50 × 10⁻⁶
Mn	0.607483	0.001860	0.004339	-	-
Fe	2.707309	0.001658	0.003868	-	-
Co	0.005203	0.007433	0.017344	-	-
Ni	0.148974	0.003192	0.007449	5.81 × 10⁻⁵	1.36 × 10⁻⁴
Cu	0.398393	0.004268	0.009960	-	-
Zn	2.545156	0.003636	0.008484	-	-
Se	0.001971	0.000169	0.000394	-	-
Al	5.826743	0.002497	0.005827	-	-
Ba	0.054625	0.000117	0.000273	-	-
As	0.005556	0.007937	0.018519	3.57 × 10⁻⁶	8.33 × 10⁻⁶
Cd	0.002873	0.001231	0.002873	7.51 × 10⁻⁶	1.75 × 10⁻⁵
Hg	0.000254	0.000363	0.000848	-	-
Pb	0.005061	0.000620	0.001446	-	-
TOTAL HI	-	0.0366	0.0853	-	-
TOTAL CR	-	-	-	7.15 × 10⁻⁵	1.67 × 10⁻⁴

## Discussion

Honey occupies a rare intersection between environment and diet: bees integrate inputs from soils, plants, dust and water across a defined foraging landscape into a product that is directly consumed. This makes honey a practical bioindicator matrix for identifying spatial patterns in environmental elemental signatures, while also enabling intake based evaluation of potential human exposure^1,13,20^. In Afyonkarahisar, multi element profiles reveal a consistent baseline fingerprint with localized departures that are environmentally interpretable. At the same time, translating measured concentrations into exposure metrics indicates low non carcinogenic risk at typical consumption levels and low-to-borderline cancer risk screening values dominated by a single element (Ni), which is important for interpreting “low risk” in a transparent way.

Across samples, the mineral hierarchy is dominated by K, with Fe and Zn among the most abundant trace elements—an elemental pattern widely described for natural honeys and repeatedly observed in Turkish ICP based surveys^4,21,22^. Comparable baseline hierarchies are also reported in other regional European datasets^5^, supporting the interpretation that most Afyonkarahisar samples reflect a broadly typical mineral composition rather than a uniformly impacted setting. Establishing this baseline is central to fingerprinting: it defines “background” against which anomalies can be recognized^13^.

Toxic elements were consistently low in absolute terms across the dataset, aligning with the common observation that honey often contains detectable but generally low levels of toxic elements in survey-type datasets^17,21,22^. In this context, Afyonkarahisar behaves largely like a background/rural-like province in terms of overall element burden.

A useful way to judge whether levels are “typical” or indicate local influence is to compare them to bioindicator studies designed around impacted environments. Case studies sampling honey from contaminated locations show that honey can reflect stronger elemental signals under clear environmental pressure^11^. Studies contrasting mining versus protected sites likewise demonstrate more pronounced fingerprint separation when a known gradient exists^15^. Targeted monitoring using bees and hive products in defined areas of concern further illustrates how localized pressure can be detected^1^. Relative to these impact focused contexts, the Afyonkarahisar dataset is best described as low burden overall, with spatially confined departures rather than broad contamination—an outcome consistent with honey as a screening and mapping tool rather than only an exceedance detection matrix^13,16^.

Correlation patterns help interpret which elements move together as suites. Strong co variation among K–Mg–P suggests a shared control on the macro mineral fraction, consistent with combined botanical and landscape/geochemical influences^4,12^. A coherent trace suite involving Cu–Mn–Ni and associations with Fe is compatible with coupled behaviour expected under soil–plant pathways and/or particulate inputs^6,12^.

PCA and HCA reduce the 20 element dataset into a small number of interpretable gradients and groupings. The main PCA axis behaves like an overall elemental richness/mineralization gradient (Table S5), and HCA resolves two main hierarchical branches that further subdivide into four sub-groups with distinct elemental profiles (Tables S6–S7; Fig. S6). Sub-group 1 represents a low-mineralization baseline, Sub-groups 3 and 4 show moderate enrichment with different element signatures (Hg-dominated vs. Al/Mn-dominated), and Sub-group 2 exhibits the strongest multi-element enrichment. This hierarchical pattern is consistent with the broader fingerprinting literature, where multivariate tools separate broad compositional modes from limited subsets with distinct signatures^4,13,17^.

To avoid treating clustering as purely statistical, we added a qualitative spatial screen within the 13 km foraging buffers (Fig. 2) using contextual cues visible in Google Earth Pro, particularly the major road network intersecting or bordering some buffers. This interpretation is intentionally hypothesis-generating rather than a formal land-use classification, but it helps anchor the clustering pattern in observable landscape context.

Within Main Group B, Sub-group 2 (samples 20 and 32, Hasankarağaç) stands out as the most elementally enriched sub-group, whose foraging buffer is intersected by a major transport corridor (Fig. 2). These samples show the strongest joint elevation (z-scores) in Pb, Zn, Co and Cd (Table S7). This co-occurrence is consistent with the broader understanding that traffic-related and other local anthropogenic activities can elevate element loads in dust and soils, which can then be reflected in bee products^6,11,18^. Importantly, we present this as a screening interpretation rather than definitive apportionment; confirmatory source attribution would require complementary matrices (soil/vegetation/pollen; dust/water) as recommended in bioindicator frameworks^1,13^.

From a consumer exposure perspective, translating concentrations into intake based metrics indicates a uniformly low non carcinogenic risk under the assumed daily consumption scenario (30 g day⁻¹ for adults; 20 g day⁻¹ for children). All element specific THQs were far below 1 and the combined hazard index remained low (HI = 0.0366 for adults; 0.0853 for children). Importantly, the HI is driven by a limited subset of elements: arsenic and cobalt were the dominant contributors in both age groups (together accounting for about 41% of HI), followed by mid level contributions from Cu, Zn and Ni (with Al also contributing). This contribution profile is useful because it clarifies that the low overall HI does not reflect uniformly negligible contributions across all elements; rather, it reflects that even the leading contributors remain well below unity under conservative screening assumptions, consistent with many published honey risk assessments that report low cumulative non carcinogenic risk at realistic consumption levels while noting children as the more conservative scenario^7,10,19,20^.

For carcinogenic risk screening (Cr, Ni, As and Cd), the element wise pattern is even more concentrated: nickel overwhelmingly dominates the total lifetime cancer risk estimate in both groups. Specifically, CR_total was 7.15 × 10⁻⁵ for adults and 1.67 × 10⁻⁴ for children, with Ni contributing around 81% of CR_total in each case; Cd is the second contributor (approximately 10%), followed by As (around 5%) and Cr (about 3%). The child CR_total lying close to (and slightly above) the commonly applied 10⁻⁶–10⁻⁴ screening interval should therefore be interpreted as a conservative, Ni driven screening signal rather than evidence of a broad multi-element carcinogenic concern. This interpretation is aligned with the way screening level cancer risks are discussed in environmental health applications, where the identity of the dominant contributor is central to interpreting marginal exceedances of heuristic ranges^8,20^.

These results should also be read in light of the conservative nature of the exposure assumptions used for screening. Risk estimates scale linearly with ingestion rate; therefore, assuming daily consumption (30/20 g day⁻¹) intentionally yields higher EDIs than intermittent intake. The child scenario is further conservative due to the lower body weight assumption. In addition, lifetime cancer risk screening implicitly assumes long term exposure, and—because element speciation was not determined—risk calculations for elements such as chromium and arsenic necessarily rely on conservative default toxicological parameters that may overestimate risk if less toxic chemical forms predominate^8,20^. Taken together, these choices bias risk upward; consequently, the conclusion of low non carcinogenic risk remains robust, while the near upper bound child CR_total is best interpreted as a protective screening outcome primarily driven by Ni under conservative assumptions^7,10,19^.

Viewed alongside Turkish surveys and international bioindicator studies from known impact settings, Afyonkarahisar honeys most closely resemble a background/rural like province in terms of overall element burden, while still exhibiting a localized fingerprint suggestive of a specific local influence (Hasankarağaç branch). This is a hallmark of honey’s bioindicator value: it can identify “where to look next” even when food safety risk remains low at typical intake^1,13,16^. A practical next step for source attribution would be targeted co sampling (soil/vegetation/pollen; dust/water) within the most distinctive foraging buffers (especially Hasankarağaç), consistent with established bioindicator approaches^1,13^.

## Conclusion

This study shows that Afyonkarahisar honeys carry clear and interpretable elemental fingerprints that are informative for both environmental insight and consumer safety. Across the 19 apiary areas sampled (*n* = 40), the mineral profile was consistently dominated by potassium, with iron and zinc as the most abundant trace elements—an overall pattern that aligns with the expected mineral hierarchy of natural honeys and supports the use of these elements as part of a regional compositional baseline.

Chemometric evaluation strengthened the bioindicator message. After log-transformation and standardization, PCA (PC1–PC2 = 56.9% variance) and Ward.D2 clustering resolved two main hierarchical groups and four sub-groups, ranging from a low-mineralization baseline to progressively higher elemental levels, with the most enriched sub-group characterized by jointly elevated standardized levels of multiple elements—most notably Pb, Zn, Co and Cd, together with higher standardized signals for Mg, Ba, Cu, Ca, P and Ni—indicating that elemental variability within the province is not uniform and that honey can sensitively flag localized geochemical and/or environmental influences. In practical terms, the fingerprints suggest that most apiary zones share comparable background conditions, while a limited subset shows an enrichment pattern that could be prioritized for follow‑up environmental screening using complementary matrices (e.g., soil, plant, dust or water).

From a public-health perspective, the data provide strong reassurance under the assessed consumption scenarios. Toxic elements were detected at low concentrations overall. Estimated exposure metrics remained well within safe margins: all THQ values were < 1, and the combined non-carcinogenic risk was low (HI = 0.0366 for adults; HI = 0.0853 for children), indicating that adverse non-carcinogenic effects are unlikely at typical intake levels. Lifetime carcinogenic risk estimates for Cr, Ni, As and Cd were low overall (CR_total = 7.15 × 10⁻⁵ in adults; 1.67 × 10⁻⁴ in children) and within or close to the commonly used tolerability range (10⁻⁶–10⁻⁴). These cancer-risk calculations also reflect conservative assumptions (notably, element speciation was not determined), meaning they are best interpreted as protective screening estimates.

Overall, Afyonkarahisar honey emerges as both (i) a useful bioindicator of fine-scale environmental variability and (ii) a safe food product with low element-related health concern under the evaluated intake scenarios. The elemental baseline and the localized enrichment signal identified here provide a focused starting point for future monitoring efforts and for geographically informed quality characterization of regional honeys.

## Supplementary Information

Below is the link to the electronic supplementary material.

Supplementary Material 1

## Data Availability

The data supporting the findings of this study are available from the corresponding author upon reasonable request.
