# Supplementary material for: Honey from Afyonkarahisar (Türkiye) exhibits low toxic-element burdens and distinct spatial elemental patterns
Source: Sci Rep. 2026 Jun 3;16:19180. doi: 10.1038/s41598-026-55009-7 (PMC13282390; doi:10.1038/s41598-026-55009-7)
Supplement: Supplementary file 1 — Supplementary Material 1 [file 41598_2026_55009_MOESM1_ESM.docx]

*Supplementary Material*

**Honey from Afyonkarahisar (Türkiye) exhibits low toxic-** **element burdens and distinct spatial elemental patterns**

**Content List**

**Table S1.** Sampling locations, sample numbers, and coordinates…………………………….2

**Table S2.** Analytical performance characteristics of the ICP-MS method for multi-element determination in honey samples……………………….…………………………………….…3

**Table S3.** Toxicological reference values (RfD and oral slope factors) used for the health risk assessment of element exposure……………………………………………………………….3

**Table S4.** Descriptive statistics of macro, trace, potentially toxic, and toxic element concentrations in the analyzed samples (n = 40)………………………………………………4

**Table S5.** Table S5. Raw loadings and standardized loadings of elements obtained from principal component analysis (PCA). Standardized loadings represent the correlation between each element and the respective principal component (loading × SD)………………………...5

**Table S6.** Composition of hierarchical clusters (Ward.D2, k = 4)…………………………….5

**Table S7.** Sub-group-wise mean standardized (z-score) elemental profiles obtained by hierarchical cluster analysis (Ward.D2, k = 4)…………………………………………………6

**Fig. S1** Spearman correlation matrix of elemental concentrations in honey samples**.**………………………………………………………………………………..………7

**Fig. S2** Macro elements in honey samples. Sample‑wise distribution of K, Na, Mg, Ca, and P concentrations shown as a stacked bar chart (n = 40, mg kg⁻¹)…………………………………8

**Fig. S3** Trace elements in honey samples. Sample‑wise distribution of Fe, Zn, Cu, Mn, Co, Ni, Cr, and Se concentrations shown as a stacked bar chart (n = 40 mg kg⁻¹)…………………..….9

**Fig. S4** Potentially toxic elements in honey samples. Sample‑wise distribution of Al, Ba, and Sn concentrations shown as a stacked bar chart (n = 40 mg kg⁻¹)……………………………10

**Fig. S5** Toxic elements in honey samples. Sample‑wise distribution of As, Cd, Hg, and Pb concentrations shown as a stacked bar chart (n = 40, mg kg⁻¹)……………………….……...11

**Fig. S6** (a) Average silhouette width for k = 2 to 6 (Ward.D2, Euclidean distance). Both k = 3 and k = 4 yielded the highest scores (0.311 and 0.310, respectively). (b) Ward.D2 dendrogram with sub-group boundaries at k = 4 indicated by coloured rectangles: Sub-group 1 (red; n = 21), Sub-group 2 (blue; n = 2, samples 20 and 32), Sub-group 3 (green; n = 10), Sub-group 4 (orange; n = 7)…………………………………………………………………………………………..12

**Table S1.** Sampling locations, sample numbers, and coordinates

| **Location Code** | **Sample No(s)** | **Location Name** |
| --- | --- | --- |
| 1 | 5, 22 | Suhut – Central |
| 2 | 13, 15, 26 | Bolvadin – Kemerkaya |
| 3 | 19, 40 | Bolvadin – Central |
| 4 | 27, 28 | Cay – Eber |
| 5 | 37, 38 | Bolvadin – Karayokus |
| 6 | 18 | Sultandagi – Camozu |
| 7 | 14, 36 | Cay – Devederesi |
| 8 | 29, 30 | Sultandagi – Yesilciftlik |
| 9 | 20, 32 | Afyonkarahisar – Hasankaraagac |
| 10 | 6, 23 | Sinanpasa – Cobanozu |
| 11 | 8, 17 | Iscehisar – Seydiler |
| 12 | 16, 33 | Sinanpasa – Central |
| 13 | 9, 25 | Bolvadin – Yaprakli |
| 14 | 10, 34 | Ihsaniye – Central |
| 15 | 7, 31 | Sultandagi – Akbaba |
| 16 | 24, 39 | Sultandagi – Yakasinek |
| 17 | 1, 4, 21 | Cay – Central |
| 18 | 2, 3, 11 | Cay – Karacaoren |
| 19 | 12, 35 | Sultandagi – Kayapinar |

**Table S2.** Analytical performance characteristics of the ICP-MS method for multi-element determination in honey samples

| **Element** | **LOD, mg kg⁻¹** | **LOQ, mg kg⁻¹** | **R²** | **Recovery**  **(%)** | **RSD %**  **Intraday** | **RSD %**  **Interday** |
| --- | --- | --- | --- | --- | --- | --- |
| Al | 0.00237 | 0.00791 | 0.9986 | 98 | 7.3 | 7.5 |
| As | 0.00017 | 0.00055 | 0.9988 | 92 | 6.1 | 6.2 |
| Ba | 0.00005 | 0.00018 | 0.9994 | 101 | 2.4 | 2.7 |
| Ca | 0.00716 | 0.02387 | 0.9992 | 94 | 2.5 | 2.6 |
| Cd | 0.00041 | 0.00137 | 0.9990 | 93 | 8.2 | 8.4 |
| Co | 0.00010 | 0.00029 | 0.9992 | 104 | 5.2 | 5.3 |
| Cr | 0.00009 | 0.00030 | 0.9996 | 96 | 5.1 | 5.2 |
| Cu | 0.00006 | 0.00022 | 0.9990 | 91 | 2.3 | 2.5 |
| Fe | 0.00104 | 0.00347 | 0.9982 | 105 | 1.8 | 2.1 |
| Hg | 0.00002 | 0.00006 | 0.9988 | 88 | 4.8 | 5.1 |
| K | 0.01917 | 0.06390 | 0.9984 | 107 | 1.4 | 1.7 |
| Mg | 0.00147 | 0.00490 | 0.9990 | 102 | 1.6 | 1.9 |
| Mn | 0.00004 | 0.00015 | 0.9993 | 94 | 2.1 | 2.2 |
| Na | 0.00228 | 0.00760 | 0.9994 | 95 | 1.1 | 1.3 |
| Ni | 0.00013 | 0.00042 | 0.9992 | 104 | 5.9 | 6.0 |
| P | 0.00752 | 0.02506 | 0.9990 | 92 | 6.6 | 6.8 |
| Pb | 0.00012 | 0.00037 | 0.9974 | 103 | 7.9 | 8.0 |
| Se | 0.00021 | 0.00071 | 0.9990 | 108 | 8.1 | 8.3 |
| Sn | 0.00159 | 0.00532 | 0.9986 | 101 | 4.5 | 4.8 |
| Zn | 0.00234 | 0.00780 | 0.9978 | 86 | 5.6 | 5.7 |

**Table S3.** Toxicological reference values (RfD and oral slope factors) used for the health risk assessment of element exposure.

| **Element** | **Category** | **RfD (mg/kg·day)** | **Oral SF (mg/kg·day)⁻¹** |
| --- | --- | --- | --- |
| Cr | Trace | 0.003 | 0.5 |
| Mn | Trace | 0.14 | - |
| Fe | Trace | 0.7 | - |
| Co | Trace | 0.0003 | - |
| Ni | Trace | 0.02 | 0.91 |
| Cu | Trace | 0.04 | - |
| Zn | Trace | 0.3 | - |
| Se | Trace | 0.005 | - |
| Al | PTE | 1.0 | - |
| Ba | PTE | 0.2 | - |
| As | Toxic | 0.0003 | 1.5 |
| Cd | Toxic | 0.001 | 6.1 |
| Hg | Toxic | 0.0003 | - |
| Pb | Toxic | 0.0035 | - |

**Table S4.** Descriptive statistics of macro, trace, potentially toxic, and toxic element concentrations in the analyzed samples (n = 40)

| **Group** | **Element** | **n** | **mean** | **sd** | **median** | **min** | **max** | **IQR** | **CV_perc** |
| --- | --- | --- | --- | --- | --- | --- | --- | --- | --- |
| Macro | Ca | 40 | 13.6049 | 8.8978 | 11.2229 | 3.5532 | 44.0594 | 9.3741 | 65.4014 |
| Macro | K | 40 | 2195.5852 | 1757.7816 | 1360.5709 | 275.2220 | 5377.3681 | 3057.9248 | 80.0598 |
| Macro | Mg | 40 | 57.3065 | 81.1102 | 32.4743 | 7.5145 | 397.5664 | 42.1943 | 141.5374 |
| Macro | Na | 40 | 23.1868 | 14.5913 | 19.8185 | 1.1065 | 83.4124 | 15.6150 | 62.9295 |
| Macro | P | 40 | 92.8055 | 46.0353 | 79.4754 | 32.5763 | 229.2021 | 61.7136 | 49.6040 |
| Trace | Co | 40 | 0.0052 | 0.0058 | 0.0038 | 0.0000 | 0.0254 | 0.0036 | 111.0417 |
| Trace | Cr | 40 | 0.0110 | 0.0140 | 0.0062 | 0.0010 | 0.0766 | 0.0062 | 127.6082 |
| Trace | Cu | 40 | 0.3984 | 0.2845 | 0.2767 | 0.0667 | 1.2030 | 0.4412 | 71.4227 |
| Trace | Fe | 40 | 2.7073 | 1.8488 | 2.4852 | 0.4089 | 7.5173 | 1.9360 | 68.2882 |
| Trace | Mn | 40 | 0.6075 | 0.5101 | 0.4466 | 0.1029 | 2.2058 | 0.6132 | 83.9627 |
| Trace | Ni | 40 | 0.1490 | 0.1510 | 0.0994 | 0.0000 | 0.7882 | 0.1487 | 101.3374 |
| Trace | Se | 40 | 0.0020 | 0.0022 | 0.0011 | 0.0000 | 0.0074 | 0.0034 | 114.0829 |
| Trace | Zn | 40 | 2.5452 | 5.8678 | 0.7294 | 0.1087 | 31.4103 | 1.1217 | 230.5475 |
| PTE | Al | 40 | 5.8267 | 10.1632 | 1.8160 | 0.5154 | 45.7144 | 2.7144 | 174.4225 |
| PTE | Ba | 40 | 0.0546 | 0.0362 | 0.0414 | 0.0136 | 0.1429 | 0.0443 | 66.2786 |
| PTE | Sn | 40 | 0.3888 | 0.5365 | 0.1120 | 0.0000 | 2.5375 | 0.7005 | 137.9818 |
| Toxic | As | 40 | 0.0056 | 0.0047 | 0.0041 | 0.0012 | 0.0209 | 0.0045 | 85.4119 |
| Toxic | Cd | 40 | 0.0029 | 0.0033 | 0.0016 | 0.0000 | 0.0134 | 0.0034 | 116.0218 |
| Toxic | Hg | 40 | 0.0003 | 0.0002 | 0.0002 | 0.0000 | 0.0007 | 0.0004 | 85.2436 |
| Toxic | Pb | 40 | 0.0051 | 0.0075 | 0.0025 | 0.0000 | 0.0346 | 0.0048 | 148.0625 |

**Table S5.** Raw loadings and standardized loadings of elements obtained from principal component analysis (PCA). Standardized loadings represent the correlation between each element and the respective principal component (loading × SD).

| **PC1** | **PC2** | **Element** | **PC1s** | **PC2s** |
| --- | --- | --- | --- | --- |
| -0.295 | 0.270 | **K** | -0.851 | 0.456 |
| -0.097 | 0.078 | **Na** | -0.279 | 0.131 |
| -0.328 | 0.009 | **Mg** | -0.947 | 0.015 |
| -0.120 | -0.469 | **Ca** | -0.347 | -0.793 |
| -0.319 | 0.154 | **P** | -0.921 | 0.261 |
| -0.231 | 0.067 | **Fe** | -0.668 | 0.113 |
| -0.226 | -0.364 | **Zn** | -0.654 | -0.615 |
| -0.325 | 0.097 | **Cu** | -0.938 | 0.164 |
| -0.271 | 0.173 | **Mn** | -0.782 | 0.292 |
| -0.267 | -0.194 | **Co** | -0.771 | -0.328 |
| -0.248 | 0.084 | **Ni** | -0.717 | 0.142 |
| -0.083 | -0.073 | **Cr** | -0.240 | -0.124 |
| -0.109 | -0.008 | **Se** | -0.316 | -0.013 |
| -0.190 | 0.091 | **Al** | -0.547 | 0.154 |
| -0.206 | -0.368 | **Ba** | -0.596 | -0.621 |
| 0.128 | -0.032 | **Sn** | 0.370 | -0.054 |
| -0.191 | -0.291 | **Pb** | -0.552 | -0.492 |
| -0.283 | 0.025 | **Cd** | -0.816 | 0.043 |
| -0.145 | -0.024 | **As** | -0.419 | -0.041 |
| -0.107 | 0.470 | **Hg** | -0.309 | 0.794 |

**Table S6.** Composition of hierarchical clusters (Ward.D2, k = 4).

| **Sub-group** | **Main Group** | **Number of samples**  **(n)** | **Sample IDs** |
| --- | --- | --- | --- |
| 1 | A | 21 | 5, 6, 9, 10, 12, 13, 14, 15, 22, 23, 24, 25, 26, 27, 28, 34, 35, 36, 37, 38, 39 |
| 2 | B | 2 | 20, 32 |
| 3 | B | 10 | 1, 2, 3, 4, 11, 18, 29, 30, 31, 33 |
| 4 | B | 7 | 7, 8, 16, 17, 19, 21, 40 |

**Table S7.** Sub-group-wise mean standardized (z-score) elemental profiles obtained by hierarchical cluster analysis (Ward.D2, k = 4)

| **Sub-group 1** | | **Sub-group 2** | | **Sub-group 3** | | **Sub-group 4** | |
| --- | --- | --- | --- | --- | --- | --- | --- |
| **Element** | **meanZ** | **Element** | **meanZ** | **Element** | **meanZ** | **Element** | **meanZ** |
| Sn | 0.307 | Pb | 3.606 | Hg | 1.242 | Al | 1.736 |
| Ca | 0.052 | Zn | 3.377 | K | 1.137 | Mn | 1.460 |
| Cr | -0.065 | Co | 3.234 | P | 0.899 | Ba | 1.253 |
| Pb | -0.137 | Cd | 3.126 | Cu | 0.825 | As | 1.041 |
| Na | -0.165 | Mg | 2.745 | Ni | 0.815 | Fe | 0.943 |
| Se | -0.187 | Ba | 2.397 | Cd | 0.790 | Cu | 0.570 |
| Ba | -0.266 | Cu | 2.395 | Mg | 0.651 | P | 0.498 |
| Zn | -0.297 | Ca | 2.382 | Mn | 0.478 | Ca | 0.496 |
| As | -0.329 | P | 1.951 | Na | 0.383 | K | 0.481 |
| Hg | -0.491 | Ni | 1.834 | Fe | 0.352 | Co | 0.461 |
| Co | -0.496 | Se | 1.392 | Al | 0.246 | Mg | 0.436 |
| Fe | -0.534 | K | 1.122 | Se | 0.141 | Zn | 0.414 |
| Ni | -0.598 | As | 0.755 | Co | 0.073 | Cr | 0.253 |
| Cd | -0.625 | Fe | 0.540 | Cr | -0.139 | Ni | 0.107 |
| Al | -0.660 | Mn | 0.520 | As | -0.189 | Na | -0.032 |
| Mg | -0.717 | Cr | 0.488 | Pb | -0.277 | Se | -0.038 |
| Mn | -0.764 | Na | -0.071 | Sn | -0.315 | Hg | -0.105 |
| P | -0.780 | Al | -0.379 | Zn | -0.341 | Cd | -0.146 |
| K | -0.808 | Sn | -0.666 | Ba | -0.798 | Pb | -0.223 |
| Cu | -0.811 | Hg | -0.683 | Ca | -0.933 | Sn | -0.281 |


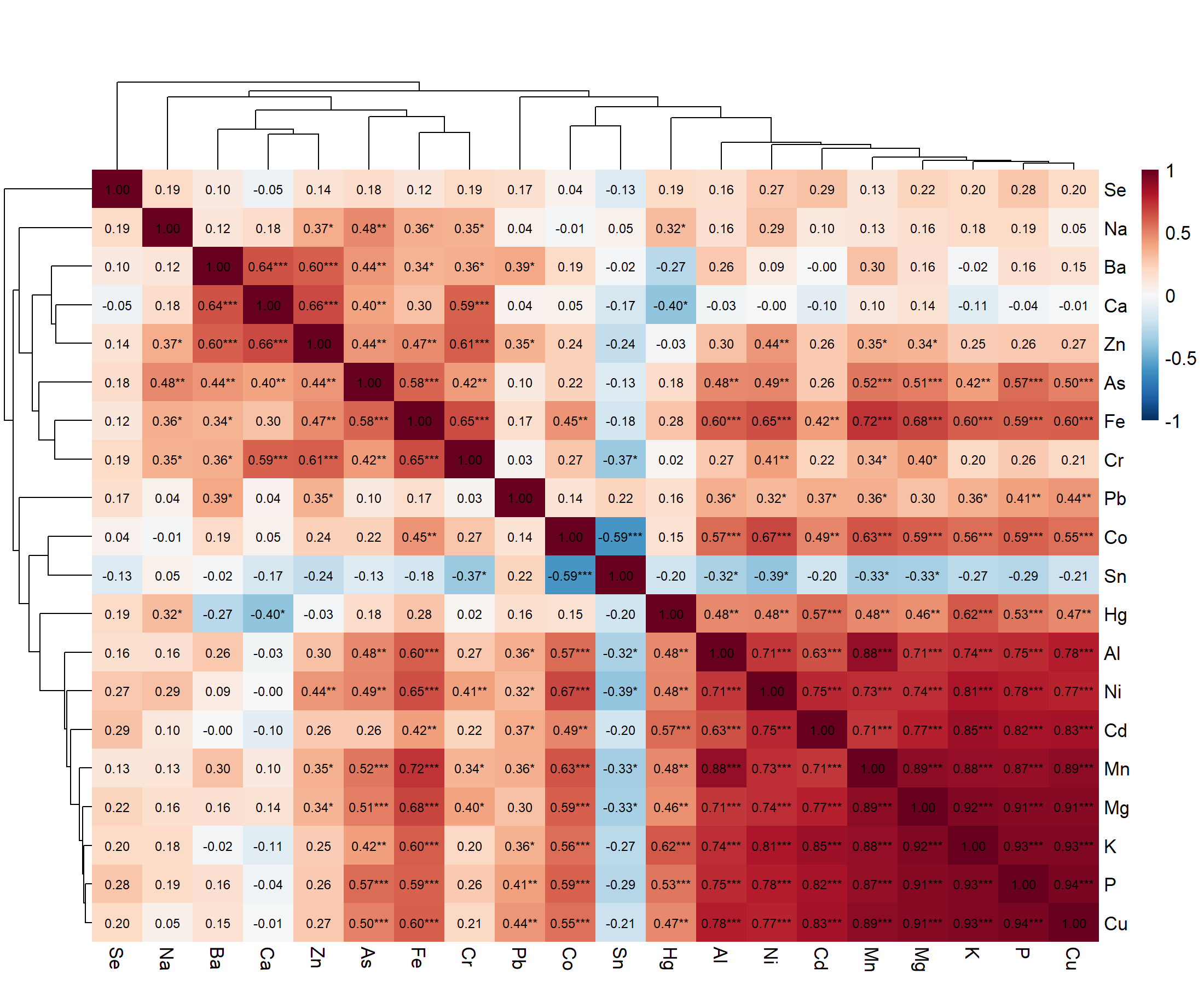


**Fig. S1** Spearman correlation matrix of elemental concentrations in honey samples. Asterisks indicate statistically significant correlations (* p < 0.05, ** p < 0.01, *** p < 0.001).

**
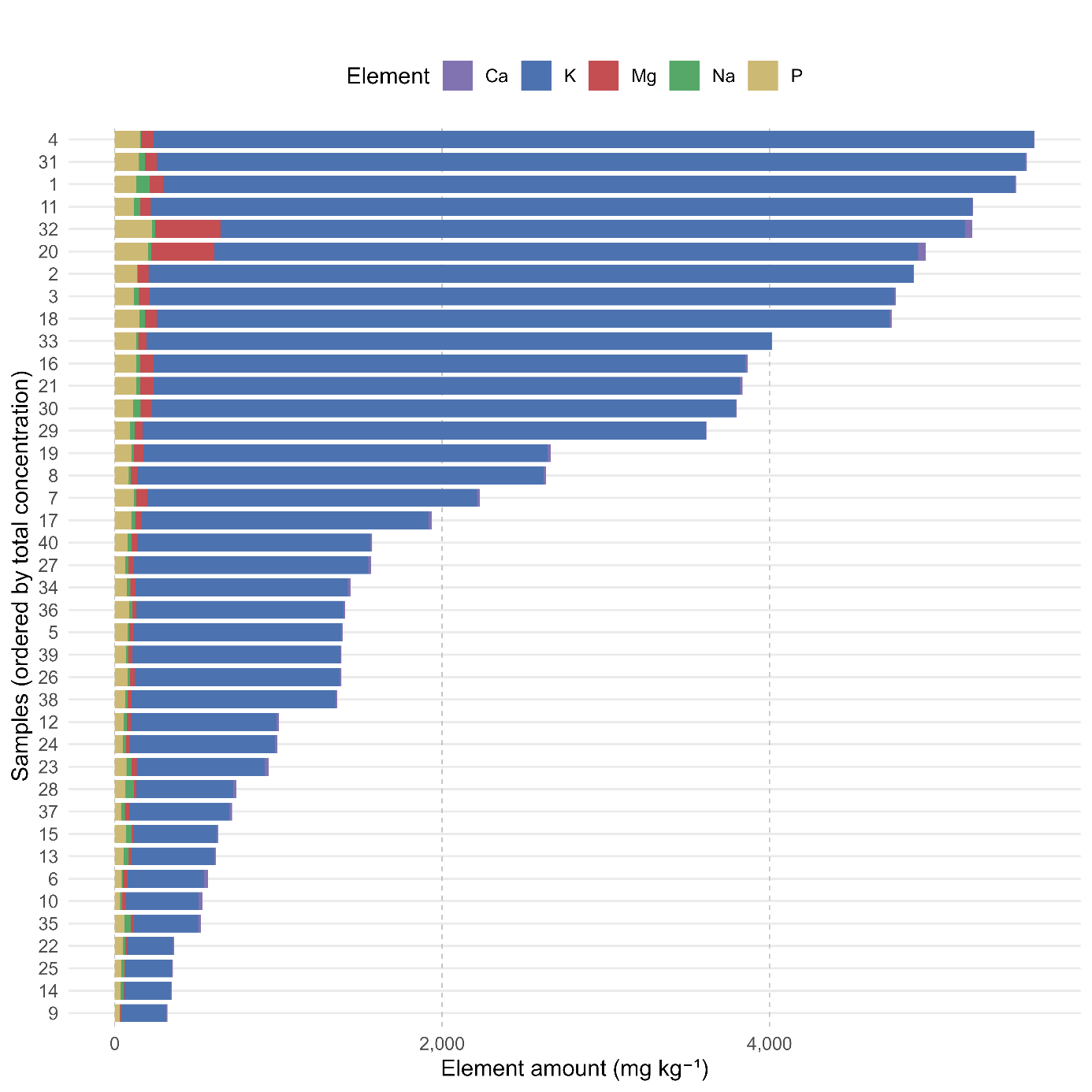
**

**Fig. S2** Macro elements in honey samples. Sample‑wise distribution of K, Na, Mg, Ca, and P concentrations shown as a stacked bar chart (n = 40, mg kg⁻¹).

**
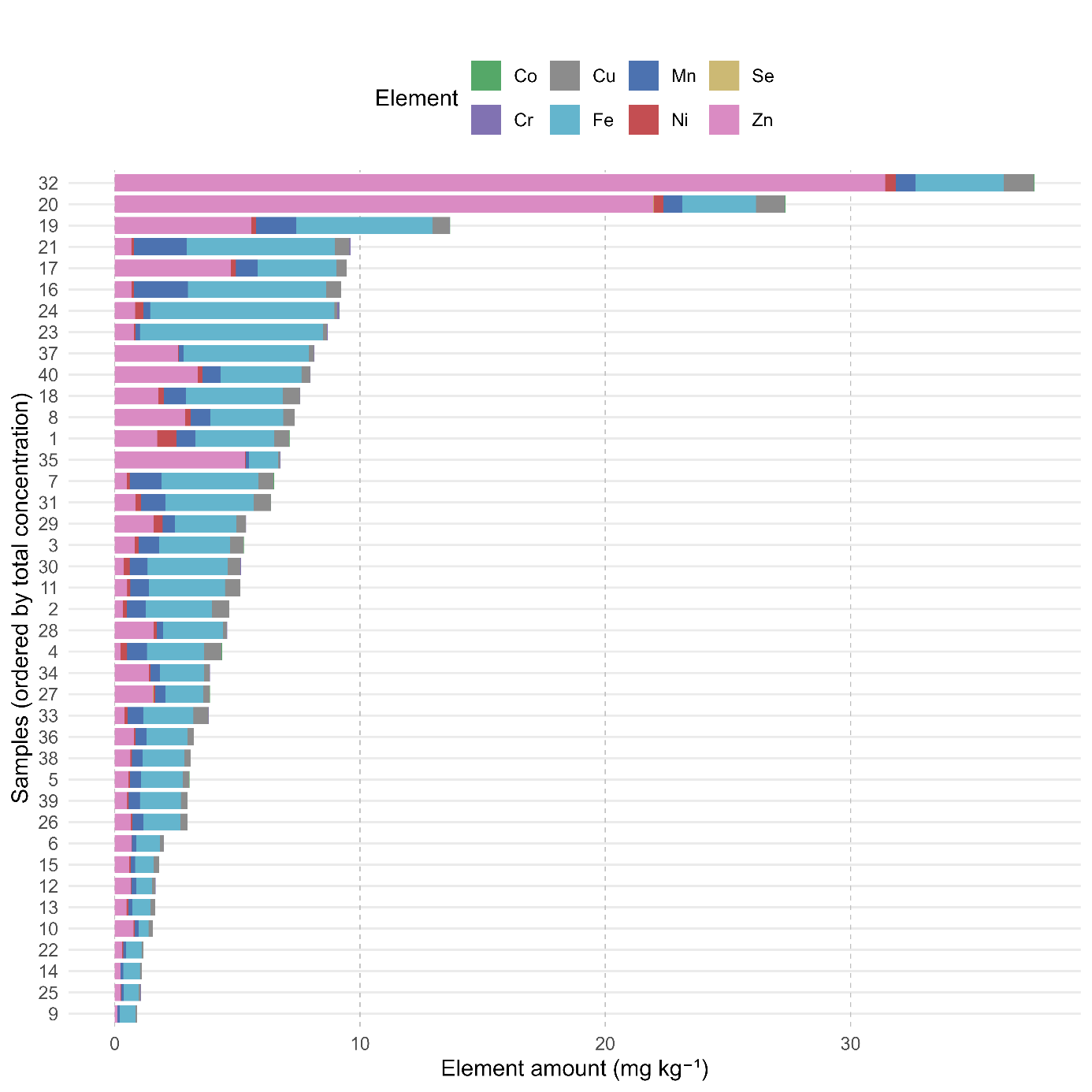
**

**Fig. S3** Trace elements in honey samples. Sample‑wise distribution of Fe, Zn, Cu, Mn, Co, Ni, Cr, and Se concentrations shown as a stacked bar chart (n = 40, mg kg⁻¹).

**
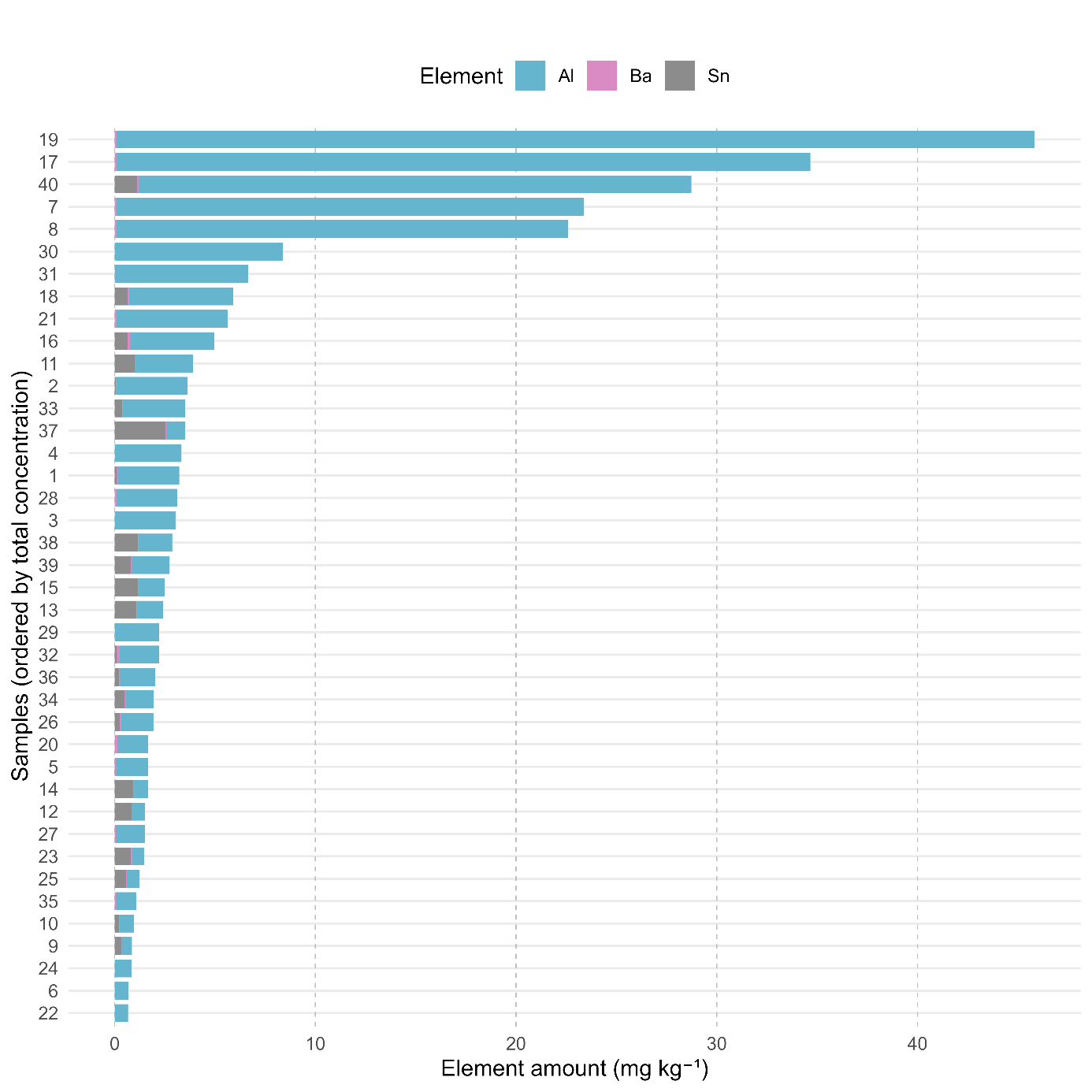
**

**Fig. S4** Potentially toxic elements in honey samples. Sample‑wise distribution of Al, Ba, and Sn concentrations shown as a stacked bar chart (n = 40, mg kg⁻¹).

**
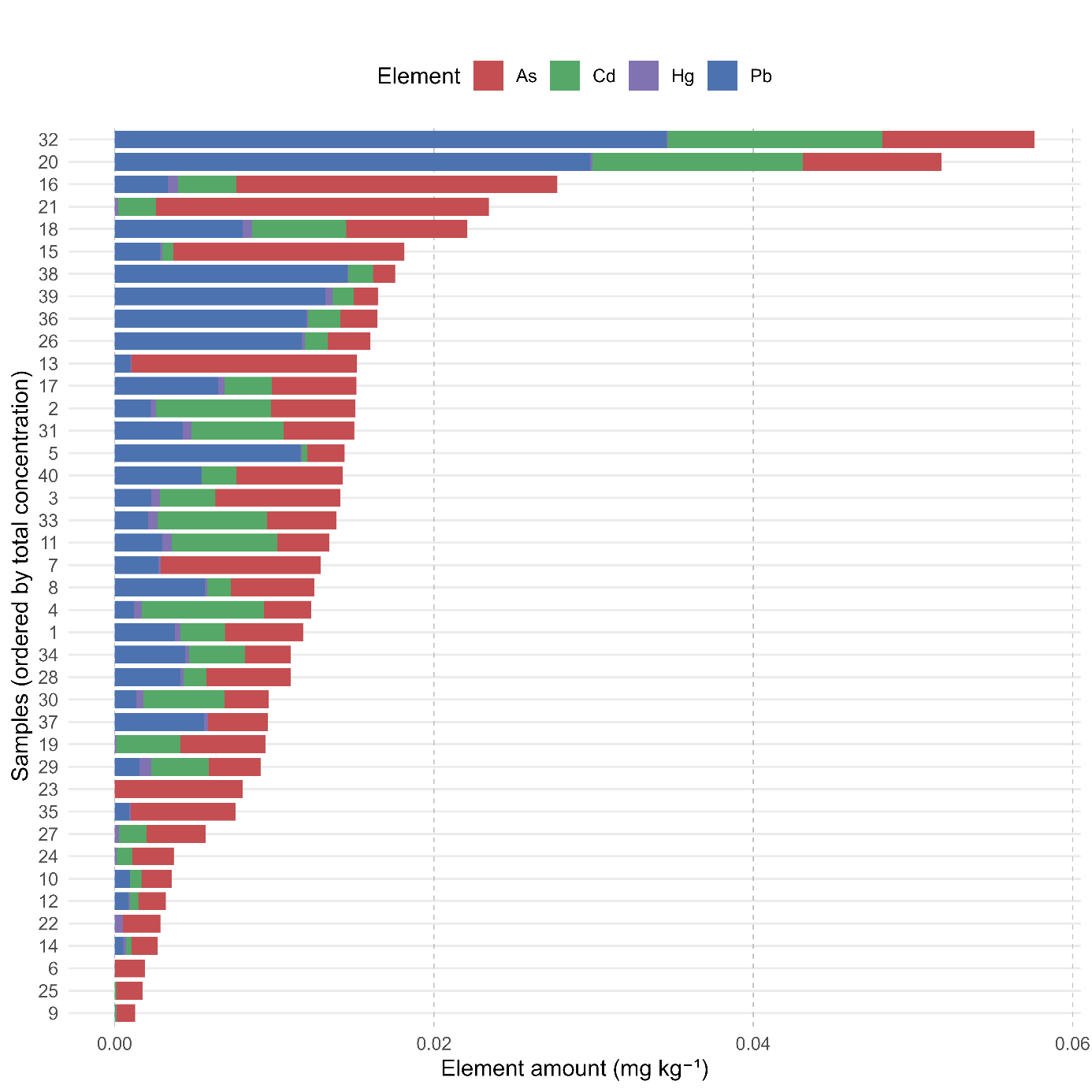
**

**Fig. S5** Toxic elements in honey samples. Sample‑wise distribution of As, Cd, Hg, and Pb concentrations shown as a stacked bar chart (n = 40, mg kg⁻¹).


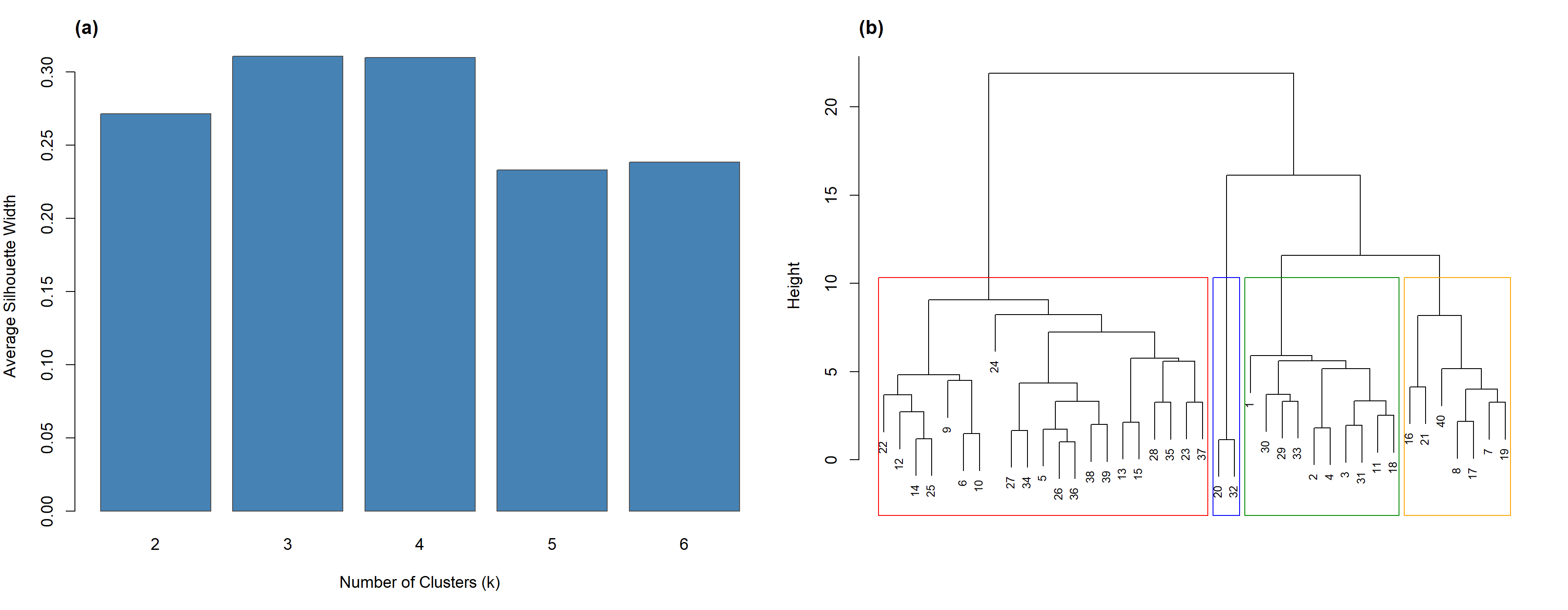


**Fig. S6** (a) Average silhouette width for k = 2 to 6 (Ward.D2, Euclidean distance). Both k = 3 and k = 4 yielded the highest scores (0.311 and 0.310, respectively). (b) Ward.D2 dendrogram with sub-group boundaries at k = 4 indicated by coloured rectangles: Sub-group 1 (red; n = 21), Sub-group 2 (blue; n = 2, samples 20 and 32), Sub-group 3 (green; n = 10), Sub-group 4 (orange; n = 7).
